# The *Drosophila* GIPC Homologue Can Modulate Myosin Based Processes and Planar Cell Polarity but Is Not Essential for Development

**DOI:** 10.1371/journal.pone.0011228

**Published:** 2010-06-21

**Authors:** Alexandre Djiane, Marek Mlodzik

**Affiliations:** 1 Department of Physiology Development and Neuroscience, University of Cambridge, Cambridge, United Kingdom; 2 Department of Developmental and Regenerative Biology, Mount Sinai School of Medicine, New York, New York, United States of America; University of Texas MD Anderson Cancer Center, United States of America

## Abstract

Epithelia often show, in addition to the ubiquitous apico-basal (A/B) axis, a polarization within the plane of the epithelium, perpendicular to the A/B axis. Such planar cell polarity (PCP) is for example evident in the regular arrangement of the stereocilia in the cochlea of the mammalian inner ear or in (almost) all *Drosophila* adult external structures. GIPCs (GAIP interacting protein, C terminus) were first identified in mammals and bind to the Gαi GTPase activating protein RGS-GAIP. They have been proposed to act in a G-protein coupled complex controlling vesicular trafficking. Although GIPCs have been found to bind to numerous proteins including Frizzled receptors, which participate in PCP establishment, there is little *in vivo* evidence for the functional role(s) of GIPCs. We show here that overexpressed *Drosophila* dGIPC alters PCP generation in the wing. We were however unable to find any binding between dGIPC and the *Drosophila* receptors Fz1 and Fz2. The effect of overexpressed dGIPC is likely due to an effect on the actin cytoskeleton via myosins, since it is almost entirely suppressed by removing a genomic copy of the *Myosin VI/jaguar* gene. Surprisingly, although dGIPC can interfere with PCP generation and myosin based processes, the complete loss-of-function of *dGIPC* gives viable adults with no PCP or other detectable defects arguing for a non-essential role of dGIPC in viability and normal *Drosophila* development.

## Introduction

Epithelial cells are polarized along their apico-basal axis through the asymmetric segregation of protein complexes. Many epithelia also show a second axis of polarization, within the plane of the epithelium and perpendicular to the apico-basal axis. For instance, this planar cell polarity (PCP) is evident in the regular arrangement of the stereocilia in the cochlear epithelia of the mammalian inner ear. Strikingly, it is apparent in (almost) all *Drosophila* adult external structures, including the body wall, where sensory bristles point posteriorly (or distally on appendages), the perfect alignment of the ommatidial units in the eye, or in the wing, where cellular hairs all point distally [1], [2]. In *Drosophila*, each adult wing cell forms one hair, an actin based process. Mutations affecting core PCP or actin cytoskeleton components result in missing or extra wing hairs [3]. This phenotype often results from a failure to localize correctly the actin polymerization nucleation center. During pupation, each cell in the developing wing becomes polarized along its proximo-distal axis and each cell produces an actin-based process. In response to the polarizing cues provided by the PCP factors, the actin polymerization nucleation center will localize to the distal side of the cell and grow distally, resulting in all the wing hairs point distally [4].

This process is under the control of both the polarity determinants that define where the wing hair grows, and the machinery *per se* that mediates the actin nucleation and polymerization. The proximo-distal polarity in the Drosophila wing is established between 12–30 hours after pupation formation (APF). It is controlled by a very well conserved PCP gene cassette used to polarize all adult structures in Drosophila (e.g. the wing, the eye, the abdomen, and the notum), and whose homologues also control PCP in vertebrates. The core PCP factors include the seven-pass transmembrane receptor Frizzled (Fz1), the scaffold protein Dishevelled (Dsh), the Ankyrin repeat protein Diego (Dgo), the 4-pass transmembrane protein Strabismus (Stbm, a.k.a. Van Gogh/Vang), the scaffold protein Prickle (Pk), and the 7-pass transmembrane Cadherin Flamingo (Fmi, a.k.a. Starry Night/Stan) [5]. This proximo-distal polarity is then interpreted in the wing to produce one actin-based wing hair that will grow distally in each cell. Some potential downstream effector genes, also referred to as tissue specific PCP genes are involved in the correct organization and growth of the wing hairs. They include classic regulators of the actin cytoskeleton such as the small GTPase RhoA, the kinase dRok, the myosin II Zipper, and its regulator Spaghetti Squash (Sqh). Other genes, such as *multiple wing hairs* (*mwh*), *tricornered*, and *furry*, are also involved in the formation and orientation of wing hairs, but their role remains less well understood [3]. Mutations in the genes required for the polarity generation typically result in misoriented wing hairs and/or more than one hair forming per cell as the actin bundle fails to form and focus at the correct spot. Mutations in the genes more directly related to wing hair formation typically give a multiple wing hair phenotype and/or deformed and split wing hairs.

To gain further insight in the control of PCP establishment we performed a gain–of-function screen in the *Drosophila* wing, and identified the GIPC homologue (dGIPC). Given the observation that the Xenopus GIPC (Kermit) binds to the C-terminal cytoplasmic tail of several Frizzled receptors including Xfz7, involved in the control of the PCP regulated process of convergent extension movements during gastrulation [6], [7], we decided to pursue the role of dGIPC during PCP generation in *Drosophila*.

GIPCs (GAIP interacting protein, C terminus) were first identified in mammals and bind to the Gαi GTPase activating protein RGS-GAIP and they have been proposed to be involved in a G-protein coupled complex controlling vesicular trafficking [8]. They all share a central protein/protein interaction PDZ domain and appear to bind to numerous membrane proteins such as Semaphorins [9],α-Integrins [10], [11], the Insulin Receptor [12], [13], the Human Leutropin Receptor [14], the β1-Adrenergic Receptor [15] and some Frizzleds [6]. GIPC also binds to Myosin VI [16] and co-localizes with Myosin VI to endocytic vesicles [17].

Even though GIPCs have been found to bind to numerous proteins including Frizzled receptors (see above), there is little *in vivo* evidence for their functional role(s). We show here that overexpressed dGIPC alters PCP generation in the *Drosophila* wing. This effect of overexpressed dGIPC is likely due to an effect on the actin cytoskeleton via the myosins, since it is almost entirely suppressed by removing one copy of the Myosin VI/*jaguar* gene. We were however unable to find any binding between dGIPC and the *Drosophila* receptors Fz1 and Fz2. The complete loss-of-function of dGIPC gives viable adults with no PCP defects arguing for a dispensable role of dGIPC in viability and normal development of the fly, including PCP generation.

## Results

### An overexpression screen for genes affecting PCP identifies dGIPC

**Figure 1:**
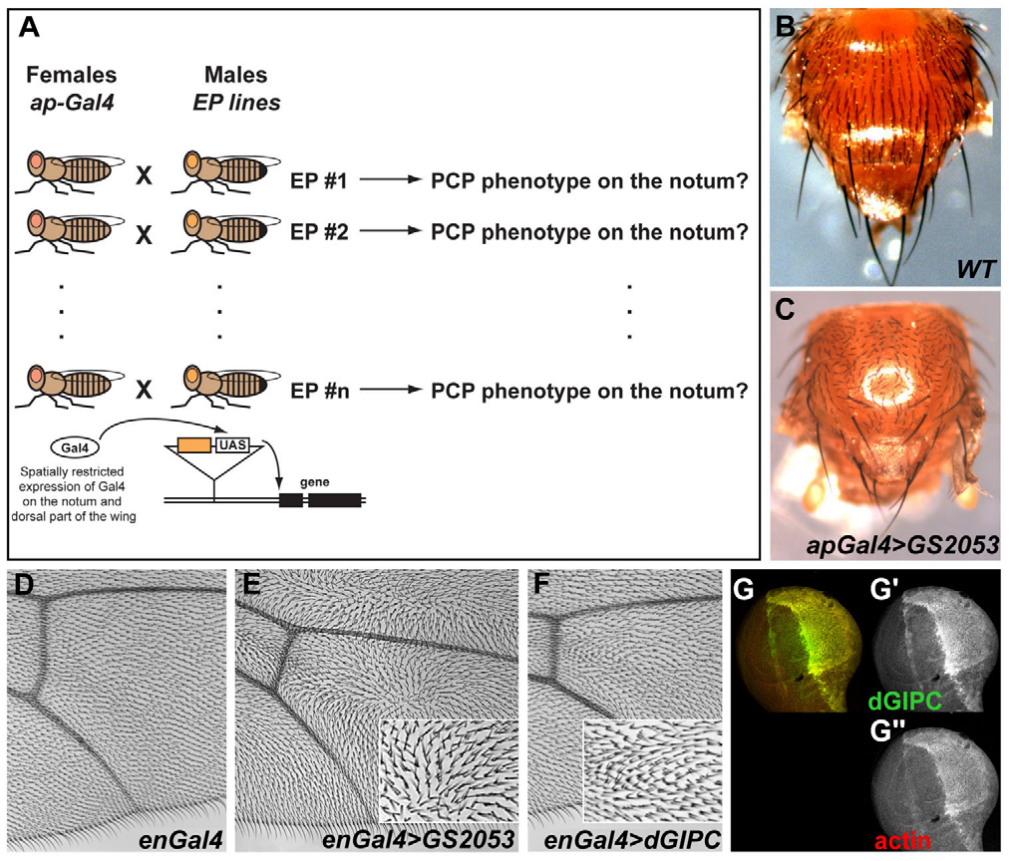
An overexpression screen for PCP defects identifies dGIPC. **A**. Schematic of the overexpression screen. Males from EP collections were mated with females expressing the Gal4 transcription factor in the apterous domain (dorsal notum and wing). **B–C**. Overexpression of the EP line GS2053 gives PCP defects in the notum (C) compared to wild type (B). **D–F**. Overexpression of dGIPC under the control of engrailed-Gal4 (F) causes PCP defects similar to that of GS2053 (E) compared to control (D). **G**. Overexpression of GS2053 under enGal4 control leads to the accumulation of dGIPC protein (green in G and G′) and of cortical actin (red in G and G″).

An intriguing aspect of PCP components, is that their overexpression gives a phenotype very similar to their loss-of-function (LOF), which could be interpreted by the fact that having a uniform or mislocalized polarity signal is as bad as having no polarity signal at all, resulting in the same loss of polarization for a given cell. We have taken advantage of this property and conducted an overexpression screen for genes potentially involved in PCP by screening EP libraries driven on the dorsal thorax by the *apterous*-Gal4 (*ap*-Gal4) driver. A similar approach has proven fruitful and led to the description of the core PCP gene *diego* [18]. Each EP line carries a transposable P-element insertion, in which the P has been modified and carries UAS Gal4 binding sites. When crossed to flies carrying a source of the transcriptional activator Gal4 expressed in a tissue specific manner, such as *ap*-Gal4, the EP line drives the expression of the gene(s) it is inserted next to (Fig. 1A) [19], [20].

Two such EP lines, EP(2)493 and GS2053 gave strong PCP defects on the notum (Fig. 1C). These EP lines also caused PCP defects in the wing when driven by *engrailed*-Gal4 (*en*-Gal4; in the posterior compartment) with wing hairs showing an impaired orientation creating swirls and waves instead of pointing distally. Furthermore, a high occurrence of multiple wing hairs was observed (Fig. 1E). No PCP defect was observed in the eye when driving these EPs with the *sevenless*-Gal4 driver (not shown) arguing that the gene associated affects only a subset of PCP events.

**Figure 2:**
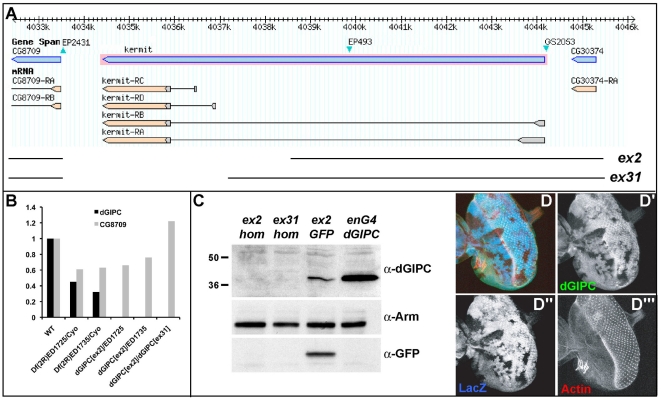
Generation of dGIPC null alleles. **A**. Schematic of the dGIPC (or kermit) locus adapted from the GBrowser from FlyBase. The blue triangles represent the mapped insertion sites of the P elements EP2431, EP493, and GS2053 used in this study. Overexpression of both EP493 and GS2053 perturbs PCP on the notum and the wing. Imprecise excision of the EP2431 recovered two dGIPC alleles, *dGIPC^ex2^* and *dGIPC^ex31^* represented by the interrupted lines. *dGIPC* mutants are viable with no obvious PCP defects. *dGIPC^ex31^* homozygous females are sterile and lay round eggs, but this is likely due to a secondary mutation, as *kermit^ex2^* homozygous females, or *kermit^ex31^*/Df(2R)1735 females show only a reduced fertility with normally shaped eggs. **B**. qPCR on ovarian extracts from different dGIPC mutant combinations. While there is no detectable dGIPC mRNA in *dGIPC* mutants, the mRNA levels of the neighboring gene CG8709 are normal. **C**. Western-blot analysis on 3^rd^ instar larval brain and discs extracts. There are no detectable dGIPC protein in homozygous *dGIPC^ex2^* and *dGIPC^ex31^* (first two lanes). Heterozygous dGIPCex2/Cyo-GFP (3^rd^ lane), and overexpressed dGIPC under enGal4 control (4^th^ lane) are shown as control. Armadillo (Arm) is used as loading control. **D**. *dGIPC^ex31^* homozygous clones in 3^rd^ instar larval eye disc marked by the loss of LacZ (blue in D and D″) have no detectable dGIPC protein (green in D and D′). There is no defect to cortical actin (red in D and D′″) in *dGIPC* loss-of-function clones.

The identified EP elements are inserted within the first intron or just upstream [19] of the single *Drosophila* GIPC homologue (*dGIPC* a.k.a. *dKermit*, *CG11546*, *l(2)02045*, Fig. 2A). *dGIPC* encodes a 336 aa protein with a single central PDZ domain, closely related to mammalian GIPCs [21], to the Xenopus Xfz3 binding protein Kermit [6], and to the Xenopus IGFR binding protein Kermit2 [13]. In order to verify that *dGIPC* is responsible for the phenotype observed, we first raised a monoclonal antibody. Upon overexpression of GS2053 driven by *en*-Gal4, a strong signal is observed in the posterior compartment of third instar wing discs proving that GS2053 drives the overexpression of *dGIPC* (Fig. 1G). Strikingly, this GS2053 overexpression induces a strong enrichment of cortical actin in these discs (Fig. 1G). Stabilization of an actin pool is consistent with the multiple wing hair phenotype seen in the adult. Second, we generated UAS-dGIPC transgenic flies in which the open reading frame of *dGIPC* is directly under the transcriptional control of Gal4 UAS sites. When overexpressed under *en*-Gal4 control, UAS-dGIPC perturbs wing hair formation with some mis-orientation and multiple wing hairs reminiscent of those observed with the GS2053 EP-line, albeit to a weaker extent, arguing possibly for some non-coding sequences affecting either the stability of the transcript or the efficiency of translation (Fig. 1E&F).

Taken together these results demonstrated that when overexpressed, the Drosophila GIPC homologue controls actin stability and affects PCP aspects of wing hair formation.

### *dGIPC* mutants generation and expression

In order to gain insight into the function of *dGIPC*, we generated null alleles by imprecise excision of the nearby P element EP(2)2431, located just downstream of the *dGIPC* ORF, between *dGIPC* and the predicted gene *CG8709* (Fig. 2A). We recovered two such small deletions, *dGIPC* ^ex2^ and *dGIPC* ^ex31^ that do not affect the *CG8709* gene but in which the entire *dGIPC* coding region is missing (Fig. 2A). In such homozygous *dGIPC* mutants, wild-type levels of CG8709 RNA are detected further indicating that in *dGIPC* ^ex2^ and *dGIPC* ^ex31^ ,the CG8709 locus and transcription are not affected (Fig. 2B). These two mutants are very likely *dGIPC* null mutants since the entire coding region is deleted in both lines based on genomic PCR, and since no dGIPC protein is detected by Western blots in homozygous third instar larval discs, or by immuno-staining in homozygous somatic clones (Fig. 2C,D).

**Figure 3:**
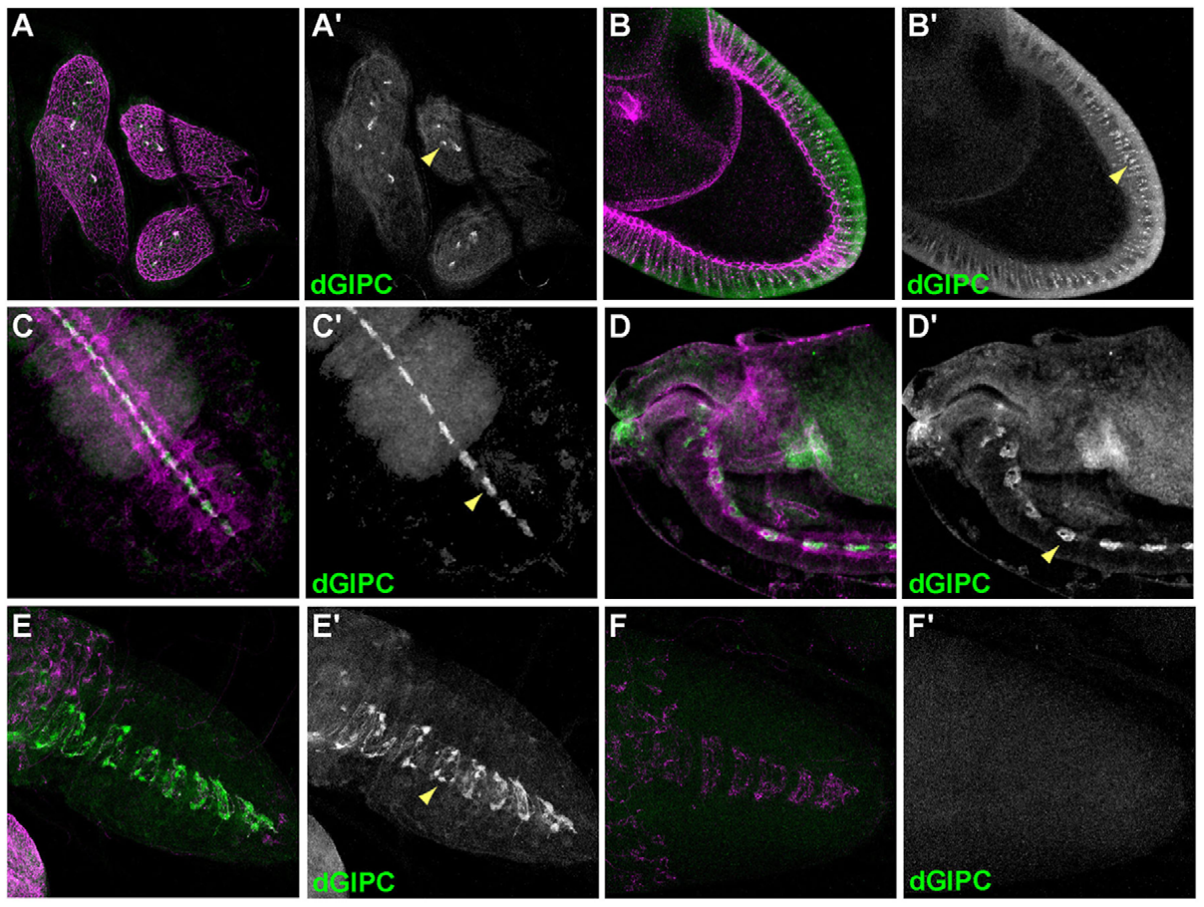
dGIPC is enriched in midline glial cells. In all panels, E-Cadherin is shown in magenta (A–F) and dGIPC in green (A–F) or white (A′–F′). **A**. dGIPC is enriched in discrete structures in 3^rd^ instar larval ganglions (yellow arrowhead). **B**. In ovarian epithelial follicular cells, dGIPC is expressed at low levels and in discrete puncta (yellow arrowhead). **C–D**. dGIPC is enriched in the midline glia in stage 16 embryos (yellow arrowheads). C: ventral view, D: lateral view. **E–F**. dGIPC is enriched in the midline glia in 3^rd^ instar larval brain (yellow arrowhead). E: wild-type larva, F: *dGIPC^ex31^/Df(2R)ED1725* null mutant larva.

The dGIPC protein is expressed in all epithelial tissue at low level, and in particular in larval imaginal discs (Fig. 2D and 3A). In the columnar follicular epithelial cells, the dGIPC protein accumulates around vesicles, suggesting that it could be involved in trafficking (Fig. 3B). Finally, high levels of expression are detected specifically in the mid-line glia both in the embryonic ventral nerve cord (Fig. 3C, D), and in the larval brain (Fig. 3E). In particular, the embryonic glial expression of dGIPC had previously been further specified as being in the anterior mid-line glia from embryos stage 13 onwards [22].

### *dGIPC* mutants do not affect wing hair formation or cortical actin

dGIPC mutants are homozygous viable and also viable over deficiencies uncovering the locus (Df(2R)ED1725 and Df(2R)ED1735). There were no wing hair defects (orientation or number) or any other noticeable defect associated with the loss of dGIPC either in homozygous animals (even when the maternal contribution was removed), or in homozygous somatic clones. In particular there were no detectable defects in epithelial cells with respect to the cortical actin (Fig. 2D and data not shown) and in midline glia where dGIPC is expressed at high levels. This argues that in *Drosophila*, dGIPC has a non essential role in contrast to what was uncovered for vertebrate GIPCs, and in particular for *Xenopus* kermit1 and kermit2 during neural crest and eye development, respectively [6], [13]. One possibility for this non-essential role of *Drosophila dGIPC* is redundancy. However, there is only one GIPC homologue in the fly, while there are at least 3 in mammals, suggesting that if there is redundancy, it is a functional redundancy with an unrelated gene rather than a structural redundancy.

The dGIPC gain of function (GOF) phenotype was a strong multiple wing hair defect (see above). Multiple wing hairs can also be generated by overexpressing core PCP proteins such as Fz. The removal of one copy of *dGIPC* did however not modify the GOF phenotype of Fz, Stbm, Pk, Dgo or Fmi, both in terms of the specific swirling patterns observed and in the number of multiple wing hairs formed. This loss-of-function study further supports the notion that *dGIPC* has no role, or at best a redundant role, in wing hair patterning and PCP generation.

### Drosophila dGIPC does not interact with Frizzled receptors

It has been reported that Xenopus Kermit1, the Xenopus homolog of dGIPC binds to a specific subset of Fz receptors (namely Xfz7 and Xfz3) and controls the Wnt1/Xfz3 mediated neural crest induction [6]. The specific pathway involved downstream of this Wnt1/Xfz3 is very likely the canonical Wnt/β-catenin pathway, although this has not been addressed in detail.

Therefore, it was possible that *dGIPC* could affect the canonical Wg pathway. However, we did not notice any Wg pathway associated phenotypes in the *dGIPC* loss of function alleles. Furthermore, we did not detect any genetic interaction between overexpressed Dfz2 and *dGIPC* either by reducing the dose of, or by co-overexpressing dGIPC with dFz2 (data not shown). We were also not able to detect a molecular association between dGIPC and the Fz1 or Fz2 Cterm cytoplasmic tails in any of the three assays employed (yeast 2-hybrid, GST pull-down, and co-IP experiments; data not shown). Finally, dGIPC does no get relocalized to the plasma membrane upon co-transfection of *Drosophila* S2 cells with Fz1 or with Fz2 (data not shown).

In light of these results, it is very unlikely that *dGIPC* plays a role at all in either the canonical or the non-canonical Fz signaling pathways.

Apart from the Frizzleds, vertebrate GIPCs have been shown to interact with a wide variety of transmembrane proteins with a carboxy end PDZ binding motif (see introduction) to promote their internalization and trafficking. Using yeast 2-hybrid, we have tested for potential interaction between full-length dGIPC, including its central PDZ domain, and a wide array of C-terminal PDZ binding motifs from *Drosophila* transmembrane proteins (usually cloning the last 60 aa). We did not detect any strong interaction with any of the following: InR (insulin) - Semaphorins 1A, 1B, 5C - EGFR (mapk pathway) - ECadherin - Notch - Dome (jak/stat pathway) - Tkv (BMP pathway) - Stbm (PCP pathway).

### The Myosin genes Zip and Jaguar control the activity of Drosophila *dGIPC*

In order to identify the genes affected downstream of overexpressed dGIPC, we have assayed for modification of the strong multiple wing hair phenotype associated with GS2053 by removing one copy of candidate genes. The results are summarized in Table 1.

**Table 1:** Genetic modification of the en-Gal4, UAS-dGIPC induced multiple wing hair/PCP phenotype.

	Allele	Modification	Source*
PCP factors	*fz^P21^*	-	P. Adler
	*fz^R52^, mwh^1^*	Enh	P. Adler
	*dsh^1^*	-	
	*dsh^3^*	-	
	*dgo^308^*	-	S. Eaton
	*dgo^380^*	-	S. Eaton
	*stbm^6^*	-	
	*stbm^X^*	-	Mlodzik-lab
	*pk-sple^13^*	-	D. Gubb
	*pk^sple1^*	Enh	
	*fmi^E59^*	-	T. Uemura
Actin cytoskeleton	*rhoA^79-3^*	-	Mlodzik-lab
	*rhoA^72O^*	-	Mlodzik-lab
	*Rac1^J11^*, *Rac2^Δ^*	-	L. Luo
	*Rac1^J11^*, *Rac2^Δ^*, *Mtl^Δ^*	-	L. Luo
	*cdc42^3^*	Enh	
	*rok^1^*	-	
	*rok^2^*	-	
	*zip^1^*	**Enh**	
	*sqh^2^*	**Enh**	D. St Johnston
	*jar^322^*	**Su**	D. St Johnston
	*fy^2^*	**Enh**	
	*fy^3^*	**Enh**	
	*mwh^1^*	**Enh**	
	*mwh^RdeMed^*	**Enh**	P. Adler
Signaling pathways	*N^55e11^*	-	
	*Dl^revF10^*	-	
	*hep^1^*	-	
	*msn^102^*	-	
	*msn^172^*	-	
	*bsk^2^*	-	
	*bsk^J27^*	-	
	*arm^4^*	-	
	*arm^8^*	-	
	*argos^rlt^*	-	
	*argos^Δ7^*	Enh	
	*Egfr^t1^*	-	T. Schupbach
	*Egfr^top-18A^*	Enh	T. Schupbach
	*rl^1^*	-	

*all stocks from Bloomington unless indicated.

Note that the enhancement seen with *fz^R52^*, *mwh^1^* is due to the *mwh^1^* allele as this enhances by itself and other fz alleles do not interact. Similarly, pk, argos and Egfr alleles only show modification with one allele suggesting second site effects.

The confirmed strong interactions are highlighted in bold and all belong to the “actin cytoskeleton” remodelers group.

We first tested for interactions with core PCP genes. No interaction was observed with the PCP mutants for *fz*, *dsh*, *dgo*, *stbm*, *pk-sple*, and *fmi*, confirming further that *dGIPC* is not involved in PCP establishment per se.

**Figure 4:**
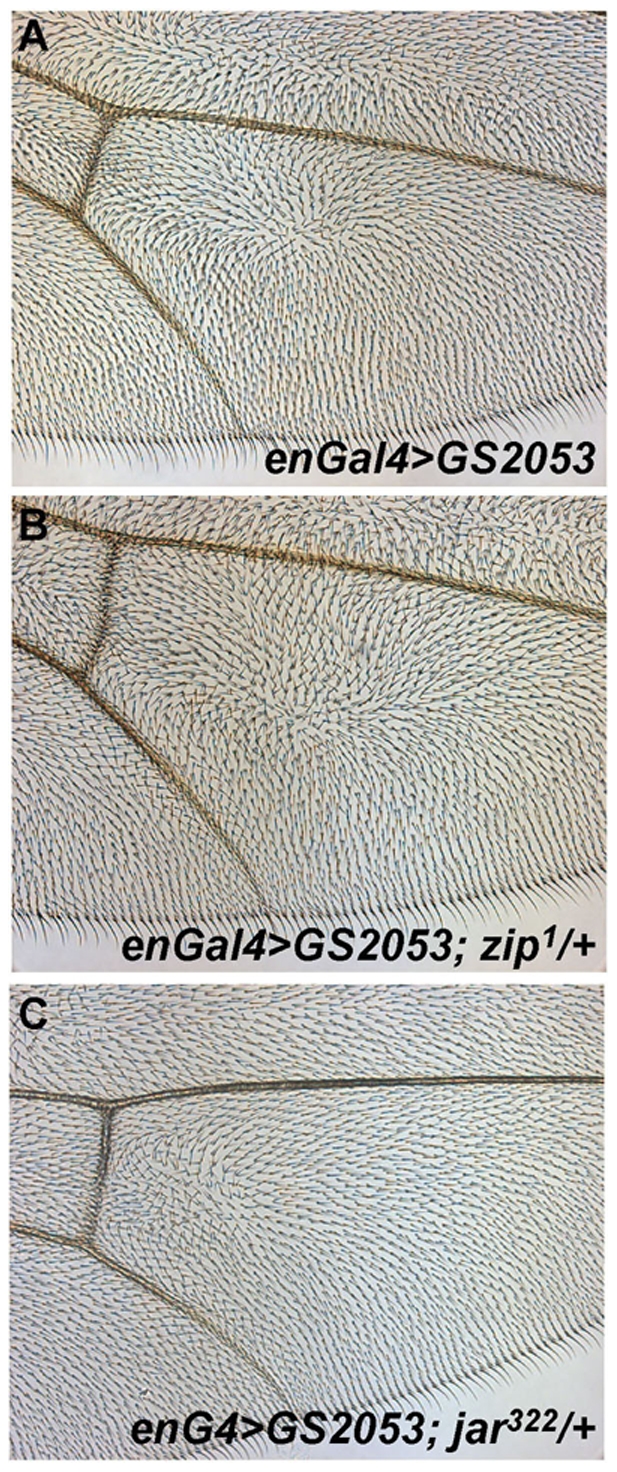
dGIPC acts through the non-muscular myosin II and myosin VI. **A**. Posterior compartment of an adult wing in which the *engrailed*-Gal4 driven overexpression of the EP line GS2053 drives the expression of dGIPC on the posterior compartment. The PCP of wing hairs is affected with swirls and multiple wing hairs forming. **B**. Removing one copy of the *Drosophila* myosin II gene *zipper*, enhances the multiple wing hair phenotype of overexpressed dGIPC (see Table 1). **C**. Removing one copy of the *Drosophila* myosin VI gene *jaguar*, suppresses the multiple wing hair and swirling phenotypes of overexpressed dGIPC (see Table 1).

We next tested for interactions with actin cytoskeleton regulators. Strikingly, an enhancement of the multiple wing hair phenotype of overexpressed dGIPC was observed when removing one copy of the small GTPase cdc42. In contrast, removing one copy of all 3 of the cdc42 related GTPases *rac1*, *rac2*, *mtl*, or of *rhoA*, and of the RhoA associated kinase *drok* did not modify the multiple wing hair phenotype of overexpressed dGIPC, indicating that the cdc42 interaction is specific. Interestingly, a dominant enhancement was also observed with *fy* and *mwh*, two genes whose mutant phenotype are multiple wing hairs. Finally an enhancement was also noted with *zipper (zip)*, the Myosin II light chain (Fig. 4B), and with *spaghetti squash* (*sqh*), the Myosin II regulatory subunit, previously implicated downstream of RhoA/dRok in the control of wing prehair numbers. The most striking results was an almost complete suppression of the overexpressed dGIPC phenotype by the removal of one copy of *myosin VI/jaguar* (Fig. 4C). Myo VI is a known partner of GIPC in mammals [16] and is involved in the control of exocytosis and vesicular trafficking [23].

These results suggest that control of the actin cytoskeleton by dGIPC is in part modulated by the non-muscular myosins MyoVI/Jaguar and MyoII/Zipper and this aspect is conserved from flies to vertebrates.

## Discussion

Using an overexpression screen for genes potentially involved in planar polarity (PCP) generation in Drosophila, we have identified the fly homologue for the PDZ domain containing gene GIPC. Overexpression of dGIPC affects PCP in the body wall and wing of the adult fly, but not in the eye. In particular, the orientation and number of wing hairs is affected. However, dGIPC null mutants are homozygous viable without any PCP defects arguing that dGIPC is not normally involved during PCP generation in the fly. Furthermore, we did not observe any obvious phenotype in homozygous null dGIPC individuals, even when the maternal contribution was removed, and even in tissues of high dGIPC expression such as the embryonic midline glia. This suggests that although GIPCs have been implicated in several important aspects of vertebrate development such as neural crest formation and head induction in the frog Xenopus laevis (kermit1 and 2 respectively) [6], [13], or such as α6β1 integrin internalization and trafficking in human endothelial cells [10], it appears that Drosophila GIPC, dGIPC, is dispensable for normal development of the fly.

This not essential role of dGIPC, could be due to redundancy, but it would be a functional redundancy, since there is only one identified GIPC homologue in the fly genome. The existence of a clear relationship between Drosophila and Vertebrates GIPCs argues for a selection to keep these genes, so that wilt type flies are fitter than dGIPC mutant flies. Thus, it could be that we were unable to uncover more subtle phenotype such as glial cell morphology or biology, or that the dGIPC function is revealed only after a challenge, absent in standard lab conditions of Drosophila culture. There are other examples of genes conserved between flies and vertebrates, that despite having well characterized roles in vertebrates, appear to have elusive functions in flies. For instance, the only p120ctn homologue in fly is not essential and homozygous null individuals are viable with no phenotype [24], [25], while the several vertebrate p120ctn homologues have well described roles in Cadherin based junction formation in epithelial cells, in neural cells and in leukocytes. A role for drosophila p120ctn, is only revealed in cells mutant for Csk, where it is involved in the removal of abnormal epithelial cells [25].

Finally, we have provided evidence that, like its vertebrate homologues, dGIPC functions with the non-muscular Myosins II and VI. Genetic interactions suggest that *myoII/zipper* inhibits dGIPC function on the actin cytoskeleton, while *myoVI/jaguar* is required to mediate dGIPC function. This antagonism between MyoII and MyoVI has previously been suggested in *Drosophila*, in particular during the asymmetric segregation of cell fate determinants in the developing neuroblasts, where MyoII is required to exclude determinants from the apical cortex, while MyoVI is required to translocate determinants basally highlighting different mode of action of these two Myosins [26], [27]. It has recently been shown that, contrary to what had been reported previously, myosin VI/jaguar is dispensable for viability in the Drosophila [28], strengthening our observation that dGIPC is not lethal. The only clear phenotype associated with Myosin VI in the fly appears to be male sterility [28], but we did not observe any male sterility in homozygous *dGIPC^ex2^* and *dGIPC^ex2^/Df(2R)ED1725* null animals, suggesting that this role of Myosin VI is independent of dGIPC.

## Materials and Methods

### *Drosophila* genetic experiments

An EP screen for potential new PCP components was performed by driving the expression of several EP collections on the notum of the adult fly using the *apterous-Gal4* driver. Interesting lines were rescreened using the *engrailed-Gal4* (posterior compartment of the wing) and *sevenless-Gal4* (mainly photoreceptors 3, 4, and 7 of the developing retina) drivers. Flies carrying a UASt transgene coding for full length dGIPC were generated by amplifying by PCR from genomic fly DNA the ORF of dGIPC and cloning in the UASt vector.

Null alleles for *dGIPC*, *dGIPC* ^ex2^ and *dGIPC* ^ex31^, were generated by imprecise excision of the P element EP2431. Other *dGIPC* alleles used were the EP lines *dGIPC^GS2053^* and *dGIPC^EP493^*, and the deletions *Df(2R)ED1725 and Df(2R)ED1735*. *dGIPC* mutant clones were generated by crossing FRT42D *dGIPC^ex31^* and eyFLP; FRT42D arm-LacZ flies.

Genetic interactions were performed at 25°C assaying for modification of the multiple wing hair phenotype of engrailed-Gal4 driven overexpression of dGIPC^GS2053^ using the different alleles stated in the text or in the figures (all available from the public stock centers unless written differently).

### Biochemistry

Yeast two-hybrid, GST-Pull down, and co-immunoprecipitation experiments between full-length dGIPC and the C-terminal cytoplasmic tails of frizzled1 and frizzled2 were performed as described previously [29]. The presence of the dGIPC protein in the dGIPC mutants was assessed by Western-Blot analysis from total protein extracts of 3^rd^ instar larval heads, following standard protocols, and using mouse anti-dGIPC (monoclonal, this study; 1∶500), mouse anti-Arm (N2 7A1, DSHB; 1∶100), mouse anti-GFP (A-11120 from Molecular Probes; 1∶500).

### qRT-PCR

Total RNA from 5 adult females egg chambers was isolated by Trizol (Ambion) and reverse transcribed using the M-MLV RT kit (Promega) and random hexamers (Promega). The levels of the cDNA for dGIPC and for CG8709 were quantified by real-time PCR using QuantiTec Sybr Green PCR mix (Qiagen), after normalization to the levels of the ubiquitous gene rp49. The calibration curves for each primer pair were constructed from serial dilutions of genomic DNA.

The primers used were:

dGIPC S: GACTGACGATCACCGACAAC AS: ACCATATTCTGGCCGTTGAG

CG8709 S:AGCGCAAGAACTCTTCAAGC AS: GTTGTTGTTTGTGGCACTGG

rp49 S:GTCGCCTGCGTTCTCAAGAG AS:GACAATTGAACTCGGCACTC

### Immunocytochemistry

Embryo collection, and dissection of larval brain and imaginal discs and antibody stainings were performed using standard protocols. Primary antibodies used were: mouse anti-dGIPC (monoclonal, this study, 1∶50), rat anti-LacZ (J. Wu, 1∶500), rat anti-E-Cad (DCAD2, DSHB; 1∶25), rhodhamine-phalloidin (Molecular Probes, 1∶50).
